# Improved histological fixation of gelatinous marine invertebrates

**DOI:** 10.1186/s12983-021-00414-z

**Published:** 2021-06-12

**Authors:** Dorothy G. Mitchell, Allison Edgar, Mark Q. Martindale

**Affiliations:** The Whitney Laboratory for Marine Bioscience, ﻿9505 N, Ocean Shore Blvd, St. Augustine, FL 32080-8610 USA

**Keywords:** Ctenophore, *Mnemiopsis leidyi*, Fixation, Preservation of zooplankton, Immunohistochemistry, In situ hybridization

## Abstract

**Background:**

Gelatinous zooplankton can be difficult to preserve morphologically due to unique physical properties of their cellular and acellular components. The relatively large volume of mesoglea leads to distortion of the delicate morphology and poor sample integrity in specimens prepared with standard aldehyde or alcohol fixation techniques. Similar challenges have made it difficult to extend standard laboratory methods such as in situ hybridization to larger juvenile ctenophores, hampering studies of late development.

**Results:**

We have found that a household water repellant glass treatment product commonly used in laboratories, Rain-X®, alone or in combination with standard aldehyde fixatives, greatly improves morphological preservation of such delicate samples. We present detailed methods for preservation of ctenophores of diverse sizes compatible with long-term storage or detection and localization of target molecules such as with immunohistochemistry and in situ hybridization and show that this fixation might be broadly useful for preservation of other delicate marine specimens.

**Conclusion:**

This new method will enable superior preservation of morphology in gelatinous specimens for a variety of downstream goals. Extending this method may improve the morphological fidelity and durability of museum and laboratory specimens for other delicate sample types.

**Supplementary Information:**

The online version contains supplementary material available at 10.1186/s12983-021-00414-z.

## Background

The tissues of gelatinous marine invertebrates are often difficult to fix. Thin epithelial cellular layers with complex morphologies surround thick regions of highly hydrated extracellular matrix associated with the organism’s hydroskeleton that resists penetration of fixatives. The contrasting physical properties of the mesoglea and fragile tissue layers is a long-standing, well known problem to laboratory biologists, field ecologists, and museum curators interested in the long and short term preservation of these kinds of animals [1]. This problem has also limited developmental biologists and others interested in using downstream molecular techniques to study various aspects of these animals’ biology.

We serendipitously have developed a new protocol to fix whole animals that better preserves overall morphology than existing methods and is compatible with a broad range of powerful molecular techniques. These include immunohistochemistry, small molecule labeling (such as with EdU), and in situ hybridization, techniques that are often difficult to interpret with morphological degradation. The method requires only common laboratory reagents and the commercially available consumer product, Rain-X® Original Glass Water Repellant (Illinois Tool Works, Inc). Rain-X® is a hydrophobic surface coating composed mostly of polysiloxanes and organic solvents (the SDS lists ethanol, acetone, and isopropanol) intended to be applied to automotive glass but also commonly used in laboratories to silanize glass surfaces, such as in preparing microscope slides. We hope this technique will be similarly successful in other difficult-to-fix jellies.

While previously published protocols [2–4] adequately fixed *Mnemiopsis leidyi* and other ctenophore embryos and very small juveniles for various labeling methods, total sample loss was high and morphological preservation of these delicate animals diminished rapidly in samples above ~ 250 μm in diameter. Many individual animals ruptured within minutes upon addition of the fixative, while others became unacceptably distorted or disintegrated during the subsequent fixation; finally, a number of the remaining samples would disintegrate during subsequent storage or labeling procedures. Relaxing the live samples with MgCl_2_ and embedding them in low-melt agarose before fixation to stabilize them improves outcomes but still leads to relatively high sample loss (around 50% from beginning to end of a typical experiment) and can introduce new problems with tissue orientation in the semi-solid matrix [2]. Our new protocol can adequately fix a wide range of samples (< 250 μm – at least 3 cm) substantially improved preservation of external morphology. The method is also effective with animals lacking intact epidermis used for regeneration experiments and appears to be effective with other taxa, including soft-bodied and gelatinous animals.

## Results

### Rain-X® fixation of cydippid and lobate stage *Mnemiopsis*

**Fig. 1:**
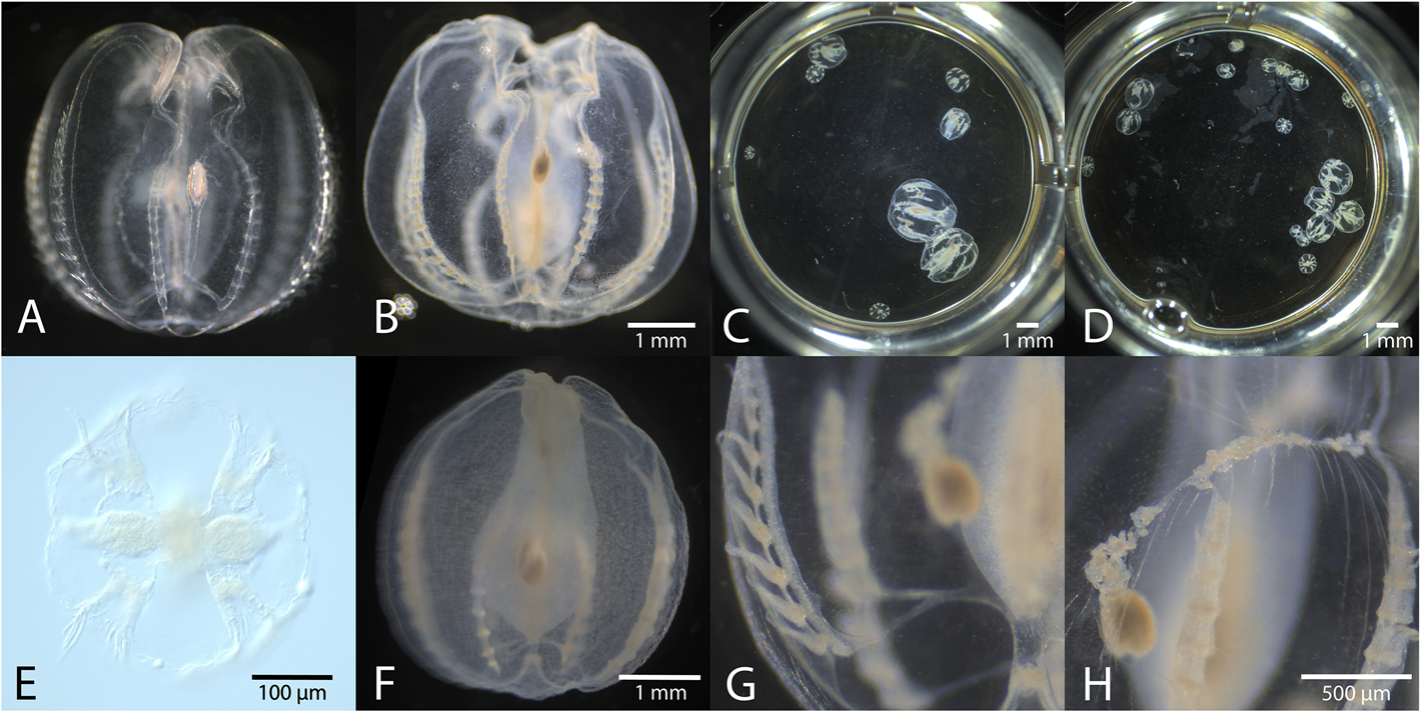
Morphological preservation of a range of *M. leidyi* samples. **a**. A live specimen of *M. leidyi*. **b**. The same animal as in A after fixation with only ~ 16% Rain-X® in filtered seawater (FSW) as detailed in protocol. Scale bar in (**b**) also applies to (**a**. **c**, **d**). A range of *M. leidyi* cydippid sizes (~ 0.5–8 mm) fixed according to the same protocol; every individual is adequately preserved across a range of body sizes. See Supplemental Fig. 1 A-C for a comparison with standard aldehyde fixation. **e**. A recently hatched *M. leidyi* cydippid (aboral view) fixed according to the modified protocol for small samples < 250 μm. **f**. A two-week old cydippid fixed according to the protocol and dehydrated into 100% methanol for long-term storage. **g**. Close-up of comb row and underlying gonad in a larger (2–3 mm) cydippid fixed according to the protocol. The aboral sense organ can also be seen in the lower right-hand corner of the image. **h**. Close-up view of tentacles of the same animal as in (**g)**. The tentacles remain outside the body and some of the tentilla (fine projections of the tentacle) remain extended. Scale bar in H also applies to (**g**)

Samples fixed with Rain-X® are consistently preserved at a range of body sizes (Fig. 1) as opposed to more standard methods such as aldehyde fixation in which a majority of samples may be shriveled or destroyed (Supplemental Figure 1 A-C). In contrast, samples treated with Rain-X® rapidly become rigid (but not hard or brittle) while retaining their prefixation shape (Fig. 1a, b). This is especially valuable for precious specimens but also serves to streamline protocols such as in situ hybridization and immunofluorescence staining since more interpretable, publication-quality images can be obtained from the same sample size. Samples remain nearly as transparent as in life unless further treated (Fig. 1g, h). We have also found that animals can be fixed with this method immediately after surgical manipulations such as amputation of the apical organ as in [3].

This fixation method is relatively forgiving. After testing varying concentrations on different sizes of animals, we settled on the concentration that allowed a range of sizes (~ 250 μm – 10 mm) to be fixed adequately with the standardized exposure time of one hour. While many individuals on the smaller end of the range are adequately fixed in a shorter time, they do not become overfixed or apparently distorted. We found that a higher concentration, ~ 37.5%, was better for larger animals (6 mm – 3 cm).

High fidelity preservation of fine structures including comb plates, tentacles, tentacle bulbs, muscles, and neural structures is apparent in these samples (Fig. 1c-g). Extremely long fixation times will eventually disrupt morphology and make the samples more brittle but we have had success with fixation for up to 3 days at 4 °C. Animals may be relaxed with MgCl_2_ before fixation if desired but even if they are not cydippids’ tentacles often remain relaxed and partially outside the body (Fig. 1h). Fixed samples do not stick to plastic so they can be stored in any convenient container such as multi-well plates or tubes, and can be stored in filtered sea water or common buffers such as phosphate-buffered saline (PBS) and stored at 4 °C, or can be dehydrated into methanol and stored at − 20 °C. The time required to fix the samples is approximately equal to or less than the time required for paraformaldehyde fixation of similar samples.

### Small-molecule labeling: EdU incorporation assay

**Fig. 2:**
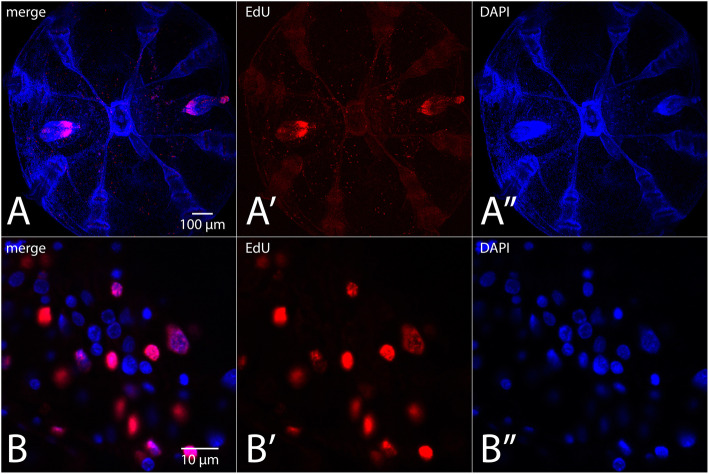
EdU incorporation assay pattern is comparable to previous published observations with improved gross morphology. A-A”. Maximum intensity projection of an aboral view of a *M. leidyi* cydippid labeled with EdU in a pulse-chase experiment as in [3]. B-B″. Higher magnification view showing co-localization of EdU label and robust nuclear staining (DAPI) in the intact epidermis of the aboral surface. EdU signal (red, A’, B′) is concentrated in the paired tentacle bulbs and in isolated cells scattered throughout the epidermis. DAPI signal (blue, A”, B″) shows that the EdU signal co-localizes with a subset of nuclei

**Fig. 3:**
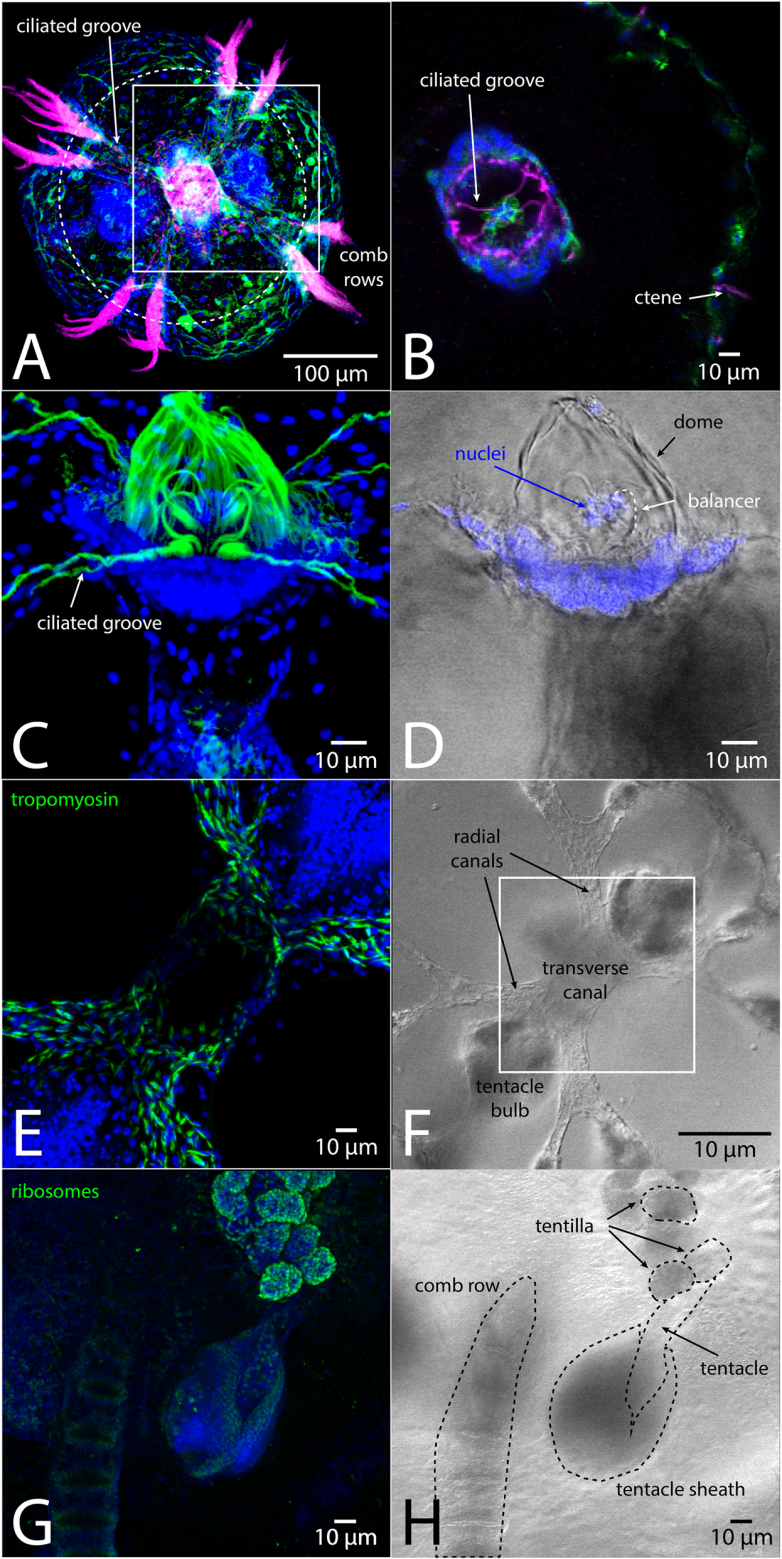
Indirect immunofluorescence confocal projections showing fine structures of *M. leidyi* cydippids preserved using ~ 16% Rain-X® in FSW. **a**. Aboral view of a whole cydippid labeled with anti-tyrosinated tubulin (magenta), anti-actin (green) and DAPI (blue). **b**. Single confocal slice representing focal plane approximated by dotted circle in (**a**), of the region indicated by the boxed area in (**a**). The slice is through the base of the aboral sense organ (apical organ). Cross-section shows preservation of fine tracts of ciliated furrows leading from comb plates into apical organ and nuclei of the epithelial floor of the apical organ itself [5]. **c**. Maximum intensity projection showing high magnification view of an apical organ labeled with anti-acetylated tubulin (green) and DAPI (blue). Balancer cilia with statolith nuclei still present are contained within the statocyst dome. Labeled ciliated furrows connect to base of each balancer. **d**. Differential interference contrast view of the same sample as C overlaid with DAPI channel from the same focal plane to highlight nuclei on balancer cilia. **e**. Aboral view of a cydippid labeled with anti-tropomyosin alpha 2 chain (green) and DAPI (blue). The smooth muscle cells highlighted line the first branches of the gut’s endodermal canal system. **f**. DIC image showing the transverse canal (infundibulum) and radial canals (first branches of the endodermal canal system through which digested particles are distributed for absorption) [6] in the same animal as E but at lower magnification. Boxed area represents panel E view. **g**. Oblique tentacular view showing tentacles emerging from the tentacle sheath. Sample is labeled with anti-eukaryotic ribosome (green) and DAPI (blue). **h**. DIC image of the sample shown in (**g**) with comb row and tentacle anatomy indicated by dashed lines. Selected coiled tentilla (finely divided projections of the tentacle) are circled

Although the mechanism of fixation by RainX® is unknown, we tested various standard molecular techniques on fixed tissue and found that most of our standard labeling techniques are compatible. We have had excellent results with small molecule labeling such as EdU incorporation assays (Fig. 2). Nuclear counter-stains such as DAPI and Hoechst work well when applied after fixation, and there is little to no autofluorescence apparent in these samples, unlike with glutaraldehyde fixation. However, fluorescently labeled phalloidin, an F-actin marker, does not work well in these samples, likely because Rain-X® contains a mixture of alcohols, which are known to reduce the binding affinity of phalloidin to F-actin. We know that this fixation method preserves F-actin by detection with F-actin antibodies (Fig. 3a).

### Larger molecule labeling: immunofluorescence assays

The overall high quality of tissue preservation following RainX® fixation allows the possibility of detailed imaging with additional molecular protocols (Fig. 3). Samples preserved following this protocol show comparable or superior results in immunohistochemistry (IHC) assays compared to samples fixed with paraformaldehyde, with minimal background or tissue distortion. After fixation, we proceed directly to the permeabilization step of our standard IHC protocol and have found that fixed samples can be stored at 4 °C in phosphate-buffered saline + 0.1% Tween-20 for at least 2 weeks before labeling with no degradation of signal.

### Nucleic acid preservation: RNA in situ hybridization

**Fig. 4:**
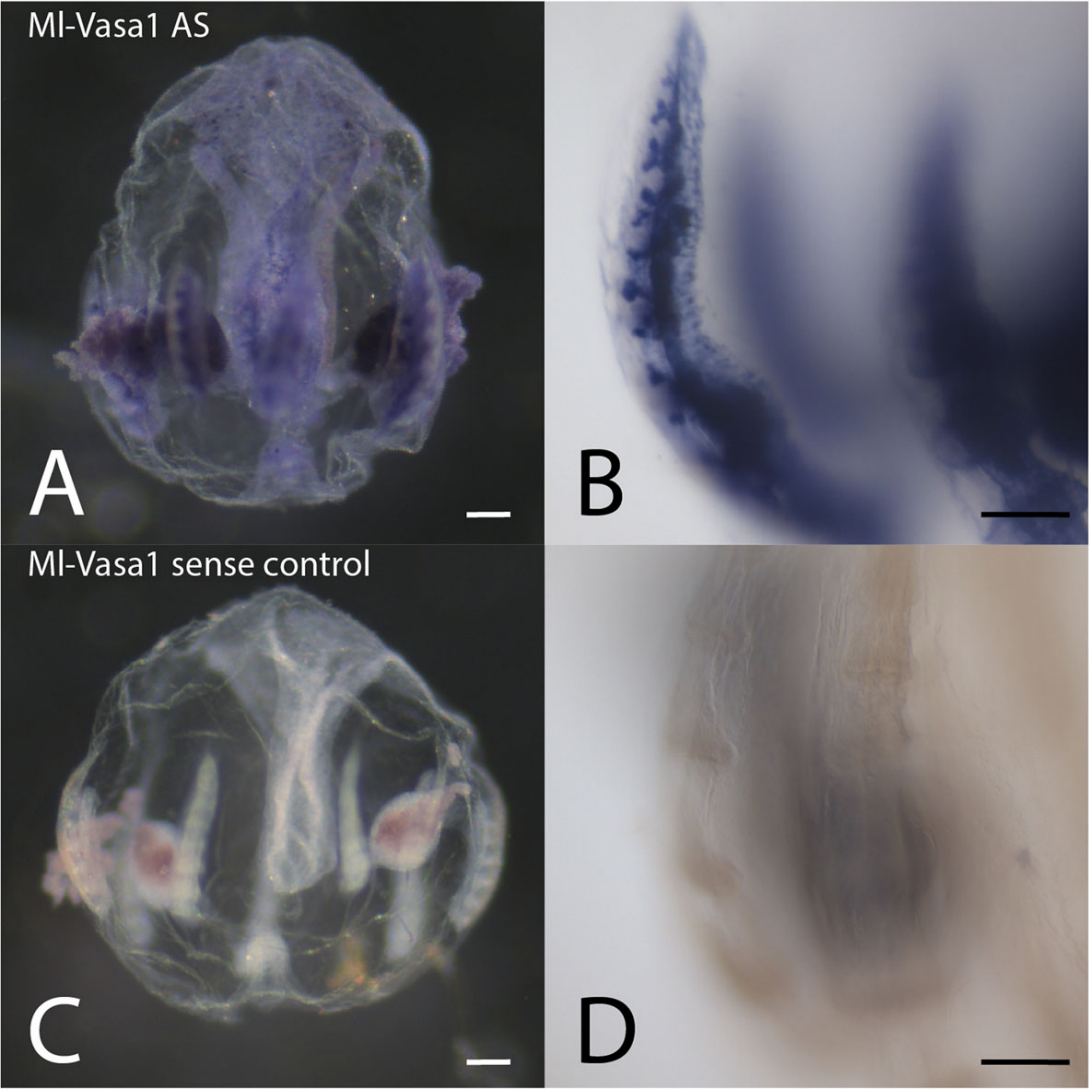
Whole mount in situ hybridization for Ml-Vasa1 following the improved fixation protocol (**a**, **b** antisense riboprobe, **c**, **d** sense control probe). Vasa has been shown to label germ line precursors in the gonads of another species of ctenophore [8]. **a**, **c**. Large (2–3 mm) cydippid stage animals viewed by compound dissecting microscope. Oral side faces up. **b**, **d**. Compound microscope views of comb rows and underlying gonad tissue. Sense control probe shows no staining in the gonad. All scale bars = 100 μm

Samples larger than ~ 250 μm fall apart during our standard whole mount in situ hybridization protocol [4]. However, fixing with Rain-X® before proceeding to our standard aldehyde fixation protocol for in situ hybridization greatly improved the morphology of larger specimens. After fixing for 1 h in ~ 16% Rain-X® at room temperature, we fixed and performed in situ hybridization following a published protocol exactly [4] using a previously validated probe for the *M. leidyi vasa1* gene [7] (Fig. 4). We were able to visualize whole-mount in situ patterns in much larger animals with superior preservation of overall morphology than previously possible in our own hands with *M. leidyi* or in published data with other ctenophore species [8, 9].

### Nucleic acid preservation: RNA and DNA extraction

We were able to extract high-quality nucleic acids, both RNA and DNA, from both juvenile and adult specimens immediately after treatment with Rain-X® using commercial, column-based kits (Supplemental File 1). The quality scores indicate that these samples would be more than adequate for input into standard molecular biology techniques including next-generation sequencing. Extracting nucleic acids from adult ctenophore tissues, especially those containing a high proportion of mesoglea, has proven challenging in the past (J.F. Ryan, D. Schultz, personal communications) and brief treatment with Rain-X® prior to lysis may actually improve nucleic acid extraction. Adult ctenophore tissue treated with Rain-X® can be dissected in a dry dish more easily than fresh, unfixed tissue. Total RNA samples extracted within 1 h in ~ 16% Rain-X® were less intact than those from fresh tissue but still considered acceptable (RIN 5.4–8.0). Genomic DNA samples collected from samples collected within 1 h of fixation were of similar quality to unfixed samples (DIN 4.5–6.4 vs DIN 4.2). Samples stored in aqueous medium for 24 h after Rain-X® fixation showed no further deterioration of RNA (RIN 6.6–7). RNA and DNA were significantly degraded in samples that had been stored in alcohol for an extended period (> 10 months) in ambient conditions after fixation with Rain-X®; it is yet unknown how rapidly degradation takes place as we have not sampled intermediate time points. Furthermore, we have not worked to optimize the storage medium, which may also be a factor. However, the successful outcome of Rain-X® with aldehyde fixation for in situ hybridization suggests that additional work to optimize this protocol for nucleic acid preservation may be fruitful.

### Application to other taxa

**Fig. 5:**
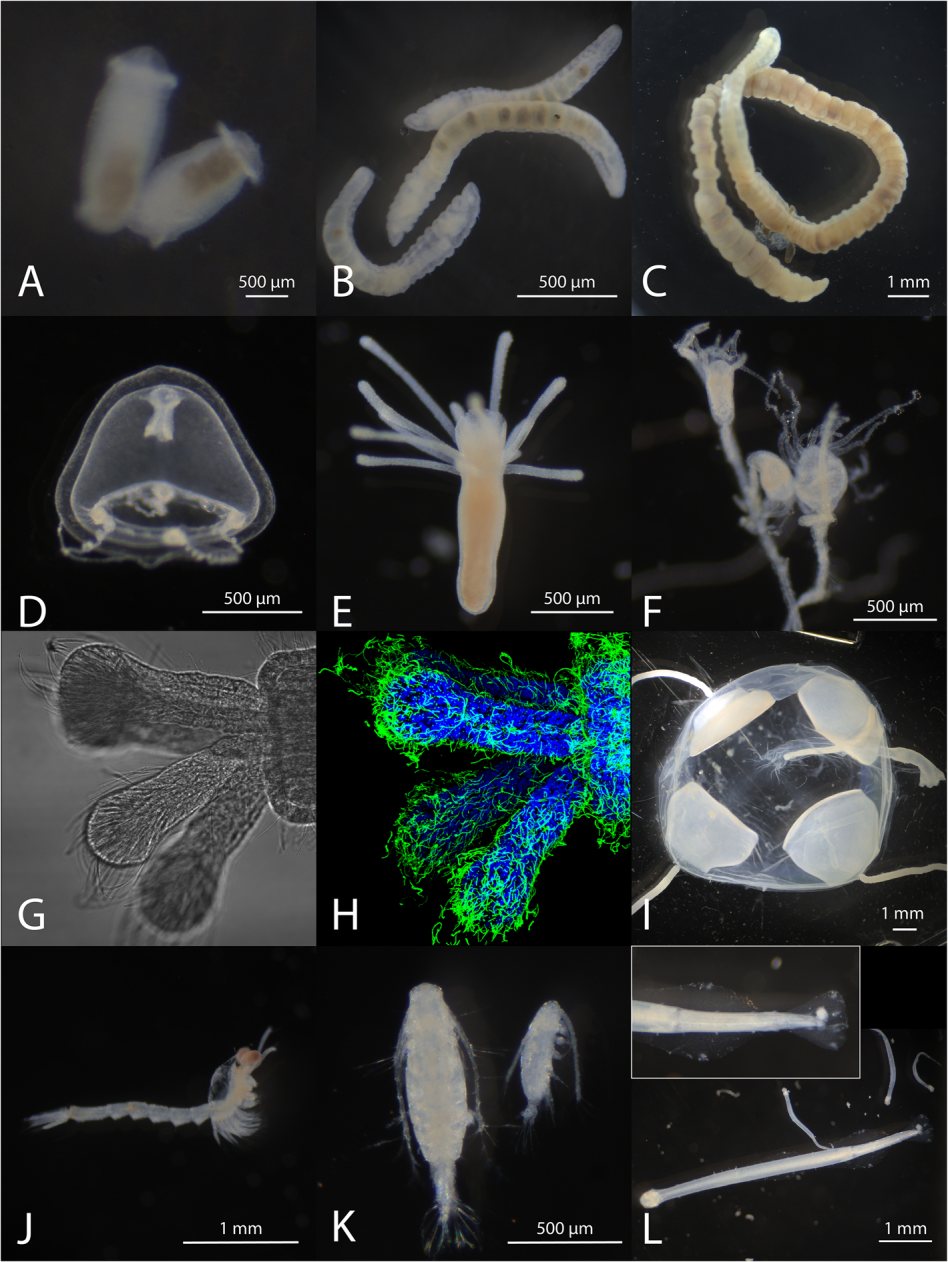
A range of gelatinous and soft-bodied animals fixed using ~ 16% Rain-X® in appropriate-strength FSW (full-strength FSW for all specimens except *N. vectensis*, which was fixed in 1/3X FSW), washed with PBST, and stored at 4 °C in PBST for 2 days – 2 weeks before imaging. **a**. Annelid worm, *Capitella teleta* adult. **b**. *C. teleta* juvenile. **c**. *C. teleta* larvae. **d**. Hydrozoan medusa. E. *Hydractinia symbiolongicarpus* primary polyp. **f**. Adult polyps of a colonial cnidarian. **g**. *Nematostella vectensis* primary polyp’s tentacles (DIC). **h**. The same sample as in (**g**) showing immunofluorescence pattern of acetylated tubulin (green) and nuclei (DAPI, blue). **i**. Hydrozoan medusa. **j**. Mysid. **k**. Copepod. **l**. Pelagic chaetognaths

While we have not optimized this technique outside of *M. leidyi*, we believe it may be useful for studying other difficult-to-fix specimens. To test this hypothesis we requested samples from departmental colleagues and performed a plankton tow off the Whitney lab dock to acquire samples of diverse marine organisms. We tested our recommended protocol based on animal size and found that all the samples we tested showed good fixation of their morphology. These samples included five cnidarians, several developmental stages of a polychaete, and a chaetognath (Fig. 5). All samples have remained intact for weeks or months stored in aqueous buffer after fixation with ~ 16% Rain-X® in species-appropriate strength FSW, even without subsequent postfixation or dehydration. Immunofluorescence performed equally well or superior to our established protocols in *Nematostella vectensis* samples.

### Potential application for museum specimens

**Fig. 6:**
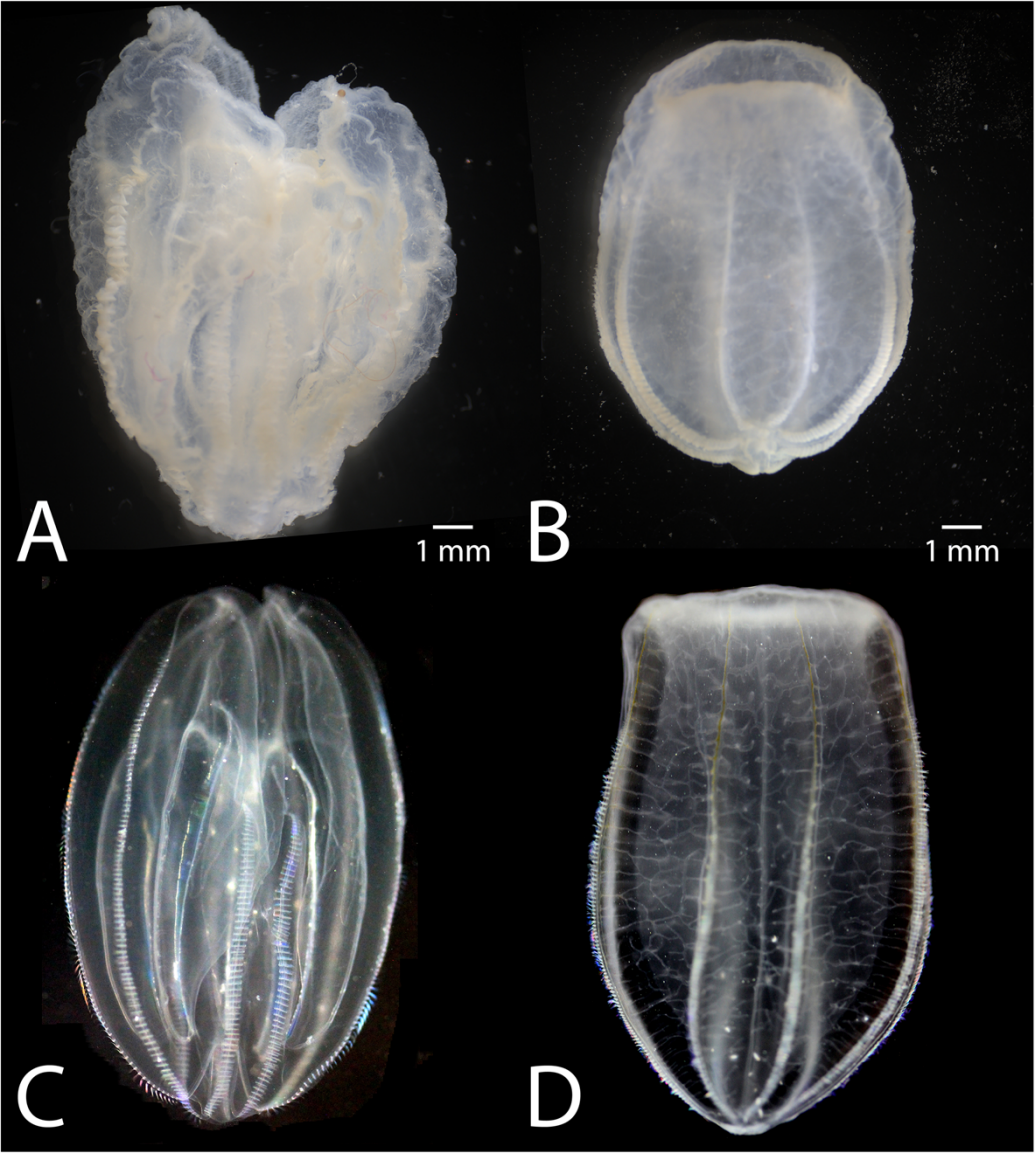
Lateral views of two species of ctenophore fixed following the novel fixation method. Oral pole faces up. **a**. *M. leidyi* lobate-stage adult after 10 months of storage at room temperature following RainX® fixation and dehydration into 80% ethanol. While some distortion is apparent, the overall integrity of the specimen is excellent. **b**. *B. ovata* adult treated identically to the specimen in panel (**a**) (fixed, dehydrated, and stored). **c**. *M. leidyi* live, lobate-stage adult for morphological comparison with the fixed sample in (**a**). **d**. *B. ovata* live adult for morphological comparison with the fixed sample in (**c**). Photographs of live samples (**c**, **d**) provided courtesy of Joseph F. Ryan and shared under Creative Commons License (CC BY 4.0)

We have not extensively optimized the method for very large (> 1 cm) samples but have performed successful proof-of-principle experiments that Rain-X® could be used to improve existing methods. Samples cannot be left in the Rain-X® mixture indefinitely. We found that after 2–3 weeks large lobate *M. leidyi* > 1 cm and other large jellyfish samples left in the solution become obviously distorted and brittle. However, fixed samples can be stored in alcohol-based or aqueous solutions. We have fixed adults of two different ctenophore species following this protocol (*M. leidyi* and *Beroe ovata*), dehydrated step-wise into 80% ethanol, and stored them on a shelf at ambient room conditions (temperature ~ 20–28 °C, contained in transparent jars on open shelving exposed to sunlight and room lighting) for ten months with no obvious signs of deterioration (Fig. 6).

We believe this preservation exceeds typical museum specimens fixed with current methods, however further optimization of these protocols will be necessary to maximize long term storage for individual species and size ranges. However, as with formalin fixation, we were not able to get good quality nucleic acids from these samples after 10 months of storage (Supplemental File 1).

## Discussion

Our accidental immersion of ctenophore tissues in the product that we normally use to prepare glass surfaces in contact with specimens for microscopic observation (RainX®) has lead to our optimization of this technique for all subsequent post fixation protocols with ctenophore specimens at all life history stages in our lab. Rain-X®, as a sole fixative or in combination with traditional fixatives, can better preserve the overall morphology of delicate biological samples such as marine jellies. We cannot present a definitive mechanism for Rain-X® as a fixative agent. The exact chemical composition of Rain-X® is proprietary (and we were unable to obtain any non-public information about its formulation), but it contains silicone polymers (polydimethylsiloxane, PDMS) partially hydrolyzed in reaction with sulfuric acid in a mixture of organic solvents and water. We were able to rule out experimentally the possibility that the organic solvents listed in the SDS for Rain-X®, individually or in combination (acetone, methanol, ethanol, or isopropanol alone or in combination with one another or water) was responsible for the high quality of the Rain-X® fixation. A different Rain-X® branded product we tested, the “2-in-1” glass cleaner formula, was not effective as a fixative agent. However, an apparently similar product from a different company, Aquapel® (PGW Auto Glass, Cranberry Township, PA, USA) appears to work similarly (Supplemental Fig. 1 D), although we have not optimized the protocol for this brand. Aquapel® is sold as a single-use ampoule with an attached applicator pad, making its format sub-optimal for our use, but we encourage researchers without access to Rain-X® Original Formula to test locally available alternative products.

PDMS is an optically clear, non-fluorescent [10], hydrophobic surfactant and its viscosity depends upon the length of the polymer. PDMS has been used to increase wetting and penetration of the herbicide glyphosate through the waxy cuticle of plants and similar compounds have been proposed as an adjuvant for drug delivery in animals [11, 12]. We speculate that a similar mechanism increases penetration of the organic solvents found in Rain-X® through tissues, permitting rapid fixation with a relatively low concentration of alcohols and/or acetone and hence superior preservation of morphology. However, it is also possible that another mechanism is involved, such as silanization of the sample itself.

## Conclusions

The addition of Rain-X® to the sample substantially improves morphological preservation of fragile samples, such as lobate ctenophores, than with prior methods. Samples treated with Rain-X® hold up better throughout permeabilization steps necessary for labeling techniques such as in situ hybridization and immunohistochemistry. In the case of immunolabeling, we find that samples fixed with Rain-X® alone are labeled as well or better than samples fixed with paraformaldehyde or post-fixed with paraformaldehyde after Rain-X®. While our preliminary trials appear to show that Rain-X® fixation alone is inadequate for long-term preservation of some aspects of samples such as nucleic acids and some pigmentation patterns, it is compatible with other fixatives and so brief pre-treatment with Rain-X® before fixation with more standard agents can improve morphological preservation while also preserving other aspects of the sample, such as preserving RNA for in situ hybridization. However, this method has some obvious exceptions, such as any aspect of the sample that might be destroyed by exposure to the known components of Rain-X® such as alcohols or acids. For example, we have not been able to preserve the native structure of fluorescent proteins or use phalloidin to stain actin with this method.

Although Rain-X® fixation might need to be optimized for different samples and sizes, we hope that this observation can benefit other labs studying a wide range of delicate marine organisms. Furthermore, the rapid and superior preservation compared with alcohols alone may be an asset for researchers interested in aldehyde-sensitive epitopes.

### Materials and methods for protocol development

#### Animal collection and care

Adult ctenophores (*Mnemiopsis leidyi* and *Beroe ovata*) were collected in the waters surrounding The Whitney Laboratory for Marine Bioscience in Marineland, FL, USA from floating docks on site, near Flagler Beach, or Anastasia Island.

Animals spawned overnight based on their endogenous circadian cycle. Juveniles were reared in 2-18 L glass or high-density polyethylene (HDPE) containers in UV-sterilized filtered seawater (FSW). Cydippids were fed primarily rotifers (*Brachionus plicatilis*, L-type, Reed Mariculture, Campbell, CA), with occasional artemia supplementation.

Known-diameter polypropylene disposable pipettes were used to measure animal size. Briefly, assorted sizes of plastic transfer pipettes were cut with razor blades and the internal diameter measured with a mini-ruler (Ted Pella, Inc., Redding, CA). Animals were transferred under a dissecting microscope using the pipettes to estimate body size.

#### EdU incorporation assay

Assays for proliferating cells were performed as in [3, 7, 13] using the Click-iT EdU labeling kit (Invitrogen Thermo Fisher Scientific, #C10424) according to manufacturer’s instructions. Briefly, live, intact cydippids ~ 1 mm in diameter were incubated in 100 μM EdU in FSW for 15 min then washed three times with 100 μM thymidine in FSW. Animals were immediately fixed according to the protocol below (i.e. 1 h in ~ 16% Rain-X in FSW at room temperature). The EdU detection reaction was performed immediately after fixation with the Alexa-567 fluorochrome included in the kit per manufacturer instructions. Nuclei were labeled with DAPI, and animals were mounted in PBST (PBS + Tween-20, see protocol) for imaging on a Zeiss LSM 710 confocal microscope.

#### Immunofluorescence assays

Immunohistochemistry was performed as in [2, 3, 7, 14]. Briefly, following fixation, samples were washed with ﻿PBS-0.02% Triton X-100 (PBT 0.02%), permeabilized with ﻿PBS-0.2% Triton X-100 (PBT 0.2%), blocked with ﻿5% normal goat serum in PBT 0.2% (blocking buffer), incubated in primary antibody diluted in blocking buffer, incubated in secondary antibody in blocking buffer, and counter-stained with DAPI; samples were washed multiple times with PBS-0.02% between each listed step. We tested animals fixed with ~ 16% Rain-X® in FSW alone and animals fixed with ~ 16% Rain-X® and postfixed with 4% PFA in FSW for 1 h at room temperature and found similar patterns with both.

Primary antibodies used include: actin (Developmental Studies Hybridoma Bank JLA20, 1:200), eukaryotic ribosomes (Developmental Studies Hybridoma Bank Y10b, 1:200), acetylated alpha-tubulin (Sigma T6793, 1:400), tyrosinated alpha-tubulin (Millipore MAB1864, 1:1000), tropomyosin alpha 2 chain (Developmental Studies Hybridoma Bank, CG1, 1:200). Secondary antibodies were Goat anti-Rat 568 (Invitrogen A11007, 1:250) and Goat anti-Mouse 488 (Invitrogen A11001, 1:250). Animals were mounted in PBST for imaging on a Zeiss M2 microscope.

#### In situ hybridization

After fixing for 1 h at room temperature in ~ 16% Rain-X® in FSW, RNA in situ hybridization was performed as in [4, 7]. Briefly, samples were post-fixed with ﻿ctenophore in situ fixation buffer (4% paraformaldehyde + 0.02% glutaraldehyde in FSW) for 5 min followed by ctenophore fixation buffer 2 (4% paraformaldehyde in FSW) for 1 h at 4 °C, washed with PBST, and dehydrated step-wise into 100% methanol and stored at − 20 °C.

Pretreatment and hybridization were performed following the published protocol exactly. In situ hybridization buffer Colorimetric probe detection was carried out using nitroblue tetrazolium/5-bromo- 4-chloro-3-indolyl phosphate. Reactions were stopped with 50 mM EDTA in sterile water and samples were cleared overnight in 80% glycerol before imaging on a Zeiss M2 microscope.

#### Nucleic acid extractions and quality analysis

Fresh and fixed material were compared in parallel. Total RNA was extracted from all samples using the Zymo Quick-RNA Miniprep (Zymo Research, #R1054) kit according to manufacturer’s instructions. DNA was extracted from all samples using the NucleoSpin Tissue Mini kit (Machery-Nagel, #740952). Sample yield and quality were determined by University of Florida’s Interdisciplinary Center for Biotechnology Research core facilities using Qubit (ThermoFisher) and TapeStation or Bioanalyzer (Agilent). Some DNA samples were only assessed for quantity by spectrophotometer (Nanodrop, ThermoFisher) due to COVID-19 epidemic-related reagent shortages.

#### Imaging

Dissecting microscope images were captured with Zeiss Stemi 508 microscope equipped with a Ximea camera system Inc. 12.4 MP camera system (MC124CG-SY-UB). Differential interference contrast (DIC) images were captured using a Zeiss Axio Imager M2 coupled with an AxioCam (HRc) digital camera using ZEN software. Fluorescent confocal images were captured using a Zeiss LSM 710 confocal microscope using ZEN software. DIC images in Fig. 3 were adjusted for contrast using a Curves Layer in Adobe Photoshop. All figures were assembled in Adobe Illustrator.

### Detailed protocols for fixation with Rain-X®

#### Materials

- Reagents
- Rain-X® Original Glass Water Repellant (Illinois Tool Works, Inc)
- Filtered seawater, UV-treated and filtered ≤1 μm (FSW)
- Phosphate-buffered saline (PBS): 8 g NaCl, 0.2 g KCl, 1.44 g Na_2_HPO_4_, 0.24 g KH_2_PO_4_ per 1 L milliQ-H_2_O, pH 7.4
- PBS + 0.1% v/v Tween-20 (PBST)
- Ethanol series: 20% ethanol in PBS, 50% ethanol in PBS, 80% ethanol

#### Equipment

- Glass spot dish (3 or 9 wells)
- 200 μl pipette
- 1000 μl pipette
- Ruler
- Mini-ruler (Ted Pella Inc. Cat.# 13,623)
- Dissecting microscope (Zeiss, Inc. Stemi 508)

#### Consumables

- Plastic multi-well plates (e.g. flat-bottom 24- or 6-well plates)
- Glass scintillation vials or other appropriate containers for larger specimens
- Plastic transfer pipettes (may be cut for desired internal diameter)
- Pipette tips
- Razor blades (to widen pipets)

#### Procedure

1. Determine appropriate volume of Rain-X® to add to the sample.
  1.1. Place the sample in enough filtered sea water that it does not touch the sides of the container. We are unaware of any constraints on the container as we have encountered no serious problems using plastic microfuge tubes, 50 ml conical tubes, plastic multi-well plates, glass spot plates, glass scintillation vials, and disposable plastic beakers, although we slightly prefer glass as samples are slightly less likely to stick. Relax animal by adding 7.5% MgCl, if desired.
  1.2. Measure the total volume of the sample (animals + sea water).
  1.3. A: Juveniles (0.25–5 mm) are adequately fixed with 200 μl Rain-X® per 1 ml sample volume (~ 16%). B: Large cydippids and small lobates (> 6 mm) should be fixed by adding 600 μl per 1 ml sample (~ 37.5%).
2. Primary fixation
  2.1. Shake the bottle of Rain-X® vigorously and withdraw the required amount in a pipet.
  2.2. Add Rain-X® directly to the sample by pipetting, with the tip of the pipet beneath the meniscus (rather than drop from above). For large samples, e.g. adults, add Rain-X® dropwise to different areas of the container and gently mix the sample by gently swirling and/or pipetting between additions. The sample might become slightly cloudy but individual animals will be visualizable throughout fixation.
  2.3. Place the sample for 1 h at ambient temperature or at 4 °C overnight (16 h) for juveniles and adults > 250 μm and small adult samples. Hatched juveniles < 250 μm are adequately fixed in 15 min at room temperature. Large adults (> 10 mm) may require extended fixation time that may need to be optimized (we have used up to 48 h at 4 °C for lobate stage *M. leidyi*).
3. Secondary fixation (optional, depending on application)
  3.1. All sizes can be secondarily fixed with common fixatives such as paraformaldehyde (PFA) without the damage to the tissue these fixatives often cause. We have successfully used 4% PFA or 4% PFA + 0.02% glutaraldehyde for secondary fixation depending on application (e.g. following the published in situ protocol for this species [4]).
4. Wash
  4.1. If possible, transfer animal(s) to a fresh well containing PBST for each wash. Rain-X® sticks to surfaces and rises to the air-water interface otherwise so transferring the sample from the middle of the container minimizes transfer of fixative.
  4.2. Wash 3 times, 10 min each, with PBST.
  4.3. After washing, check for visible droplets of Rain-X®, which can be easily identified as shiny “bubbles” adhering to the sample or container. If Rain-X® carryover is present, wash several more times. Gently pipetting the sample up and down and then allowing it to settle for 5–10 min can help to detach Rain-X® droplets from the sample.
  4.4. Transfer the sample into fresh PBST in its final storage container or the vessel that will be used for sample labeling if continuing directly to another protocol.
5. Dehydrate (optional, depending on application).
  5.1. For long-term storage, samples < 5 mm may be dehydrated into 100% methanol with a series of 20-min washes. The final 100% methanol wash should be repeated 3 times.
  5.2. For long-term storage of very large samples (e.g. whole adult ctenophores) we dehydrated slowly through a graded series of ethanol washes (20, 50, 80%) with one hour for each wash. Repeat the 80% wash twice, then perform a final 80% ethanol wash 24–48 h later. NB: other storage solutions are likely also compatible but we have not optimized any alternatives.
6. Store samples
  6.1. Samples may be stored in PBST or similar aqueous solution at 4 °C; we have stored samples for up to 2weeks with no deterioration of morphology or IHC signal.
  6.2. Samples in 100% methanol may be stored at − 20 °C.
  6.3. Larger samples in 80% ethanol may be stored at room temperature.

#### Troubleshooting

**Fig. 7:**
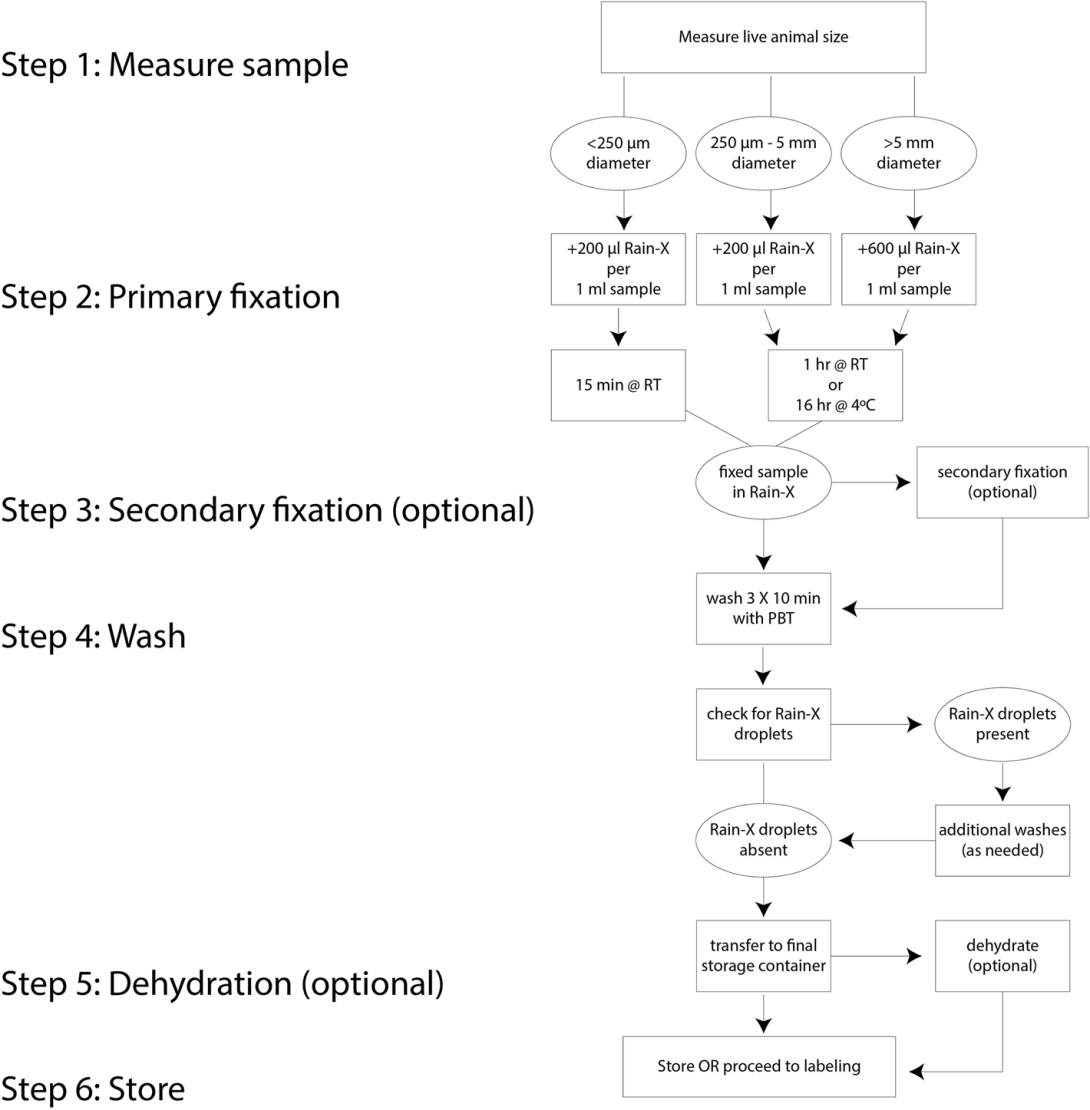
Protocol overview flow chart showing required and optional steps for fixation with Rain-X®. The protocol is described in detail below

We have optimized the protocol, including time and ratio of Rain-X® to the volume of tissue + sea water for the ctenophore *Mnemiopsis leidyi* (which is particularly difficult to fix) and found that the same protocol yielded acceptable results with *Beroe ovata*. Figure 7 summarizes the method as we use it. However, other species may require optimization of the treatment time and/or ratio of Rain-X® added to the sample. We recommend 1) starting with the values used here; 2) keeping time constant and varying concentration if optimization is needed; and 3) empirical trials of different concentrations of Rain-X®. Further modifications may be required for different sample collection goals and environments.

We found that morphology is best preserved when the samples are never dehydrated for techniques involving aqueous labeling solutions such as immunohistochemistry and EdU incorporation assays. When practical, begin the next step of the assay immediately after removal of the primary fixative and washing (Step 4). Table 1 proposes solutions for difficulties we anticipate might arise in optimizing this technique for different applications.

**Table 1:** Anticipated problems and suggested solutions in deploying the Rain-X® fixation method

Problem	Solution
Uneven Fixation, especially in larger animals	1. Pre-mix Rain-X® and FSW, then transfer animal to the mixture in the smallest volume possible or mix continuously during the addition of Rain-X®
Samples rupture or lyse on addition of paraformaldehyde or other secondary fixative	1. Extend Rain-X® treatment time before adding secondary fixative
Animals appear distorted	1. Relax animals by adding 7.5% MgCl before adding fixative, Add Rain-X® more quickly, 2. Pre-mix Rain-X® and FSW, then transfer animal to the mixture in the smallest volume possible 3. Reduce fixation time. As with any fixative, increased duration of fixation will eventually make the samples brittle and/or distorted.
Large animals appear distorted	1. Pre-mix Rain-X® and FSW, then transfer animal to the mixture in the smallest volume possible
Samples stick to plastic plates	1. Use glassware such as spot plates or scintillation vials 2. Use coated (“nonstick”) plasticware such as gelatin-coated plates or siliconized microfuge tubes
Samples do not fit into or get stuck inside transfer pipet	1. Cut tip to enlarge plastic pipet or pipet tip 2. Use polished glass, gelatin-coated Pasteur pipet
Sample initially appears well fixed but deteriorates during labeling	1. Perform washes in a larger volume 2. Use transfer pipette with wider mouth 3. Use less stringent detergent solution
Rain-X® droplets adhere to samples (usually appears as small, shiny “bubbles”)	1. Wash thoroughly after fixation 2. Add extra washes
Rain-X® droplets adhere to samples (not resolved by aqueous washes)	1. Dehydrate and rehydrate through graded alcohol series
Animals float to the top of the well and disintegrate at the air-water interface	1. Rain-X® will be disproportionally distributed across the well unless it is well mixed during the addition. Gently pipette up and down, or remove solution from the bottom of the well and gently add to the top to push the sample below the surface. 2. Try another vessel for fixation. We have found a glass spot plate to produce this effect less often than a multi-well plate.
Tissue starts to wrinkle during downstream applications	2. Keep the animals in a greater volume of water so that less is exposed to air

## Supplementary Information

**Additional file 1: Fig. S1A.** Several live *M. leidyi* cydippids. B. The same cydippids after the addition of 4% paraformaldehyde. C. Higher magnification view of cydippids fixed with 4% paraformaldehyde. D. A cydippid treated with the alternative auto glass product Aquapel® in the same way as our protocol typically uses Rain-X® as proof-of-principle that similar products may work in the same way.

**Additional file 2.**

## Data Availability

The raw data used in the current study are available from the corresponding author on reasonable request.
