# Supplementary figures and images for: Improved histological fixation of gelatinous marine invertebrates
Source: Front Zool. 2021 Jun 12;18:29. doi: 10.1186/s12983-021-00414-z (PMC8196456; doi:10.1186/s12983-021-00414-z)

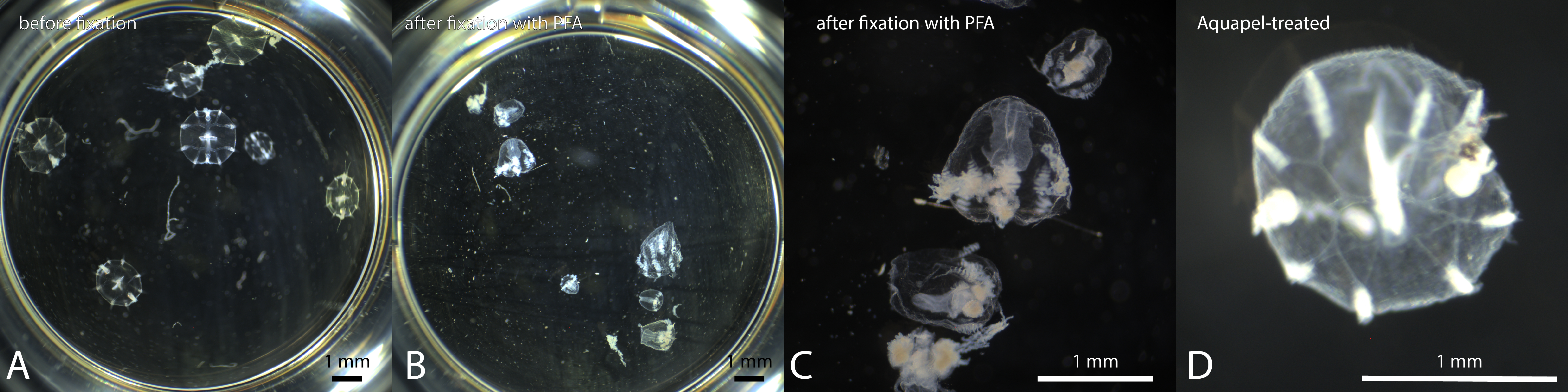

Supplement: Supplementary file 1 — Additional file 1: Fig. S1A. Several live M. leidyi cydippids. B. The same cydippids after the addition of 4% paraformaldehyde. C. Higher magnification view of cydippids fixed with 4% paraformaldehyde. D. A cydippid treated with the alternative auto glass product Aquapel® in the same way as our protocol typically uses Rain-X® as proof-of-principle that similar products may work in the same way. [file 12983_2021_414_MOESM1_ESM.jpg]
